# Novel use of a Synovis™ coupler device for a surgical lymphovenous anastomosis for the treatment of refractory chylothorax in a patient with central lymphatic flow disorder

**DOI:** 10.1002/ccr3.5636

**Published:** 2022-03-20

**Authors:** Joshua Rezkalla, Majid Husain, Ginger Slack, Sanjay Sinha

**Affiliations:** ^1^ 6915 Department of Cardiology Mayo Clinic Rochester Minnesota USA; ^2^ Division of Pediatric Cardiology Department of Pediatrics David Geffen School of Medicine at UCLA Los Angeles California USA; ^3^ Department of Plastic Surgery David Geffen School of Medicine at UCLA Los Angeles California USA; ^4^ Division of Pediatric Cardiology Department of Pediatrics University of California Irvine‐Children's Hospital of Orange County Orange California USA

**Keywords:** cardiothoracic surgery, cardiovascular disorders, congenital heart disease, critical care medicine, lymphatics

## Abstract

Refractory post‐operative chylothorax in congenital heart disease is difficult to treat. We present a case of intractable neonatal chylothorax after cardiac surgery due to central lymphatic flow disorder that was treated by creating a lymphovenous anastomosis of the thoracic duct to the left external jugular vein for definitive treatment.

## Graphical abstract

Lymphovenous anastomosis is an evolving novel treatment for complex chylothorax after cardiac surgery but requires a multidisciplinary team for planning and execution.

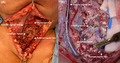

## 1 INTRODUCTION

Postoperative chylothorax and lymphatic complications in CHD are increasing in incidence as the complexity of cardiac operations also increases, with a reported incidence of 2%–5% following cardiac surgery.^1^, ^2^ It is associated with substantial morbidity and mortality and leads to longer hospital stay, increased hospitalization cost, and increased in‐hospital mortality.^1^, ^2^, ^3^ However, due to the heterogeneity of CHD and the lymphatic complications associated with it, the management of chylothorax and lymphatic disorders varies widely after conservative measures fail.^1^, ^3^, ^4^ Despite the development of new diagnostic and treatment modalities in the recent years, there is a paucity of literature in neonatal lymphatic treatment outcomes and pathways.^2^ Central lymphatic flow disorder (CLFD) is an emerging entity defined as a condition with abnormal to absent central lymphatic flow with effusions in more than 1 compartment and the presence of dermal backflow through lymphatic collaterals in the abdominal wall.^1^, ^5^ CLFD is associated with genetic syndromes and congenital cardiac diseases and often requires ventilatory support and intensive care from birth.^1^, ^5^ Treatment of CLFD is difficult, and the disease has a high morbidity and mortality. In limited case reports, LVA has been shown to be a potential treatment for these patients but has not been reported following a thoracic duct disruption.^1^, ^2^, ^5^ We present a challenging case of intractable chylothorax in CHD due to CLFD that was treated with the full gamut of lymphatic interventions and ultimately culminated in a multidisciplinary microsurgical lymphovenous anastomosis creation for definitive treatment of neonatal lymphatic complications.

## 2 CASE DETAILS

**FIGURE 1:**
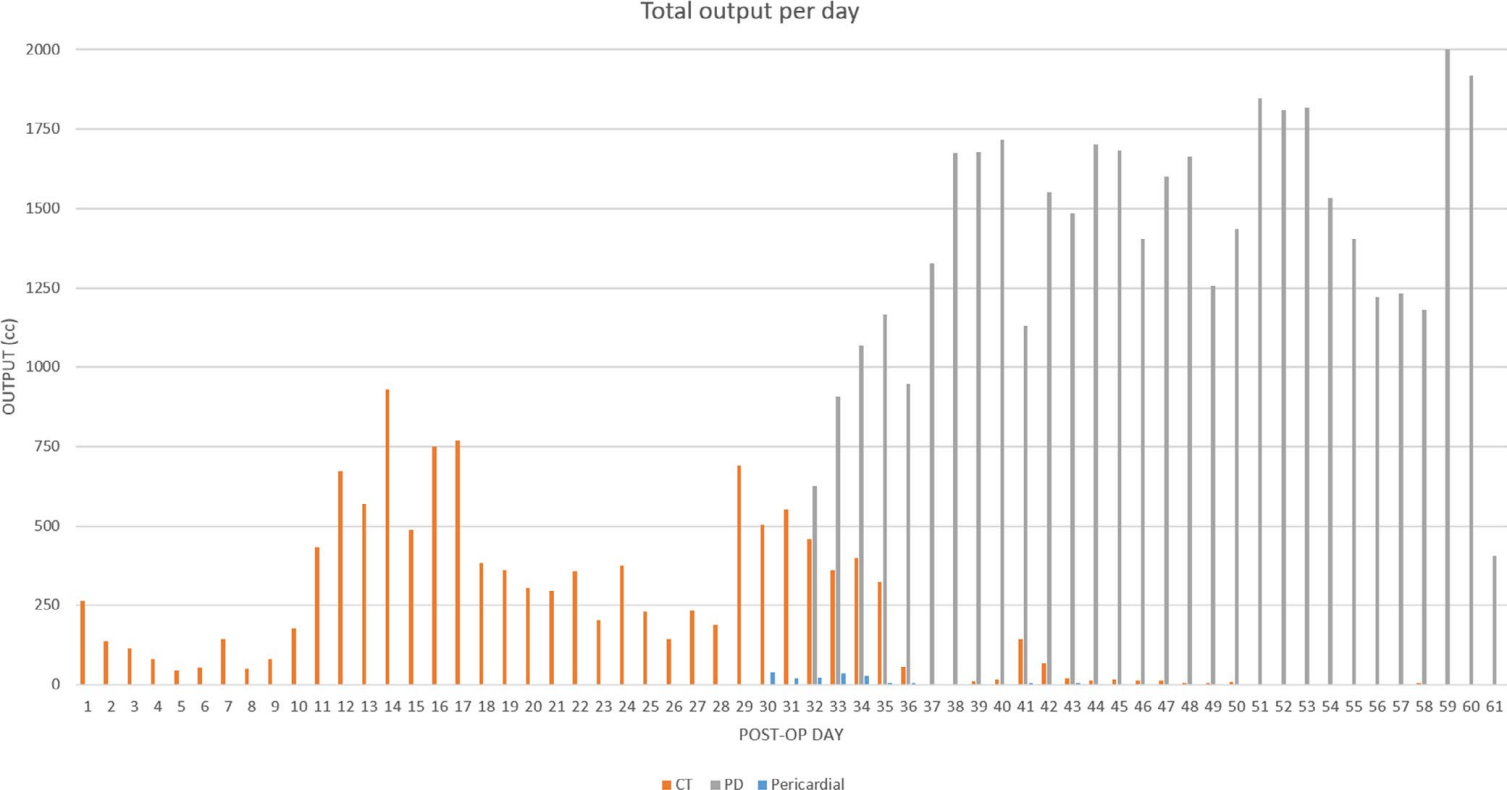
Total output per day measured postoperatively after type 2 truncus arteriosus repair. Notable time points‐POD#9: Chylous CT output noted. POD#13: Evaluation of chylothorax with diagnostic catheterization and right PA stenting with improvement in PA pressures. POD#18: Diagnostic catheterization and conventional lymphangiogram with direct thoracic duct lipiodol embolization and abdominal thoracic duct disruption.POD#30: Pericardiocentesis with the placement of pericardial drain. POD#42: Lymphovenous anastomosis.POD#61: Patient bradycardic arrest and death secondary to multi‐organ failure. *CT, chest tube drainage; PD, peritoneal dialysis drainage; POD, post‐operative day; PA, pulmonary artery

**FIGURE 2:**
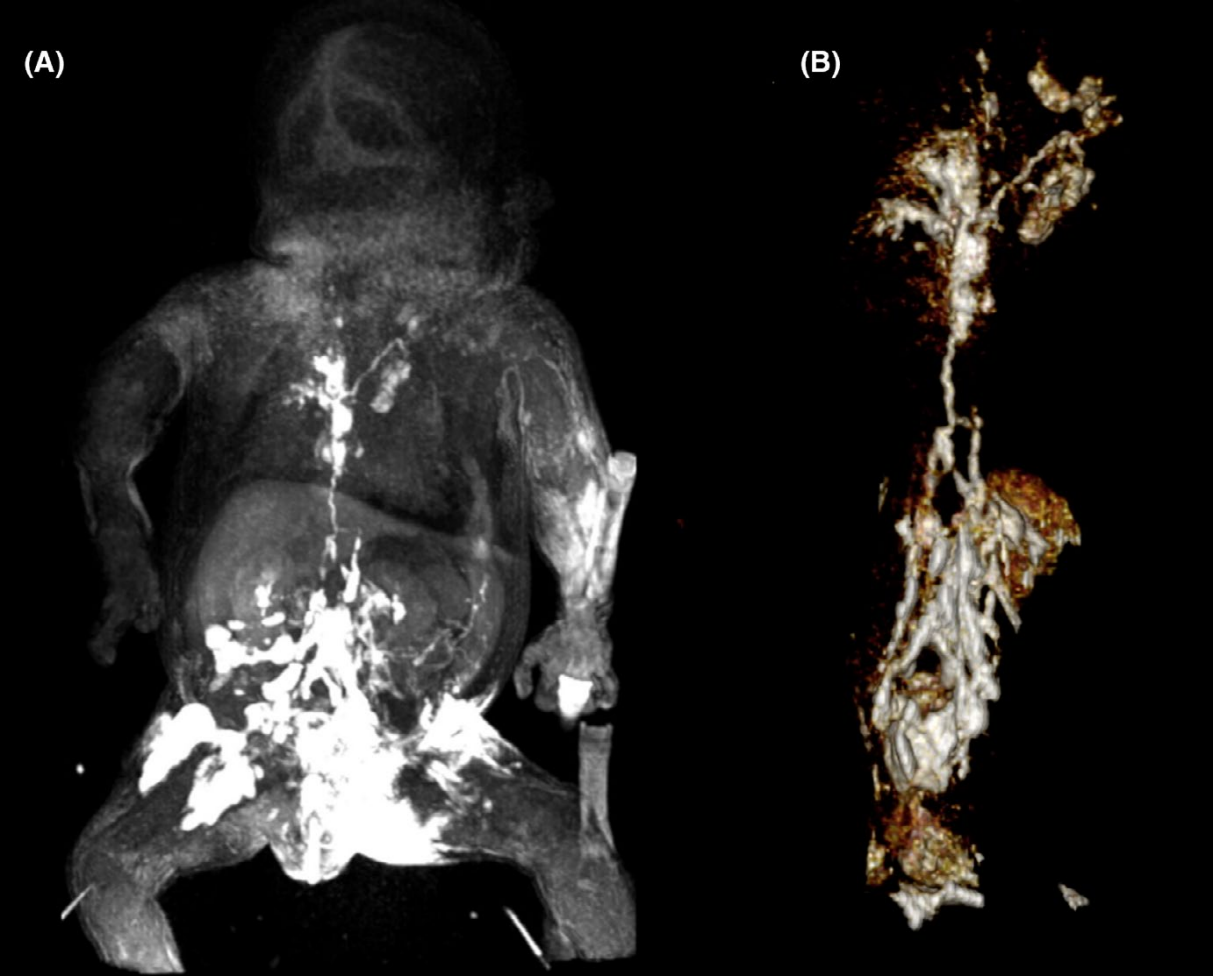
(A) Intranodal dynamic contrast‐enhanced MR lymphangiogram of the abdomen and thorax demonstrating the cisterna chyli lying left of the midline. The thoracic duct is continuous with the cisterna and lies initially to the left of the midline and then immediately crosses to the right aspect of the spine ascending along the right of the spine (with several cystic dilatations) and crossing midline at T5 to its terminus at the left subclavian/innominate vein confluence. (B) MRI 3D reconstruction of lymphatics vessels

**FIGURE 3:**
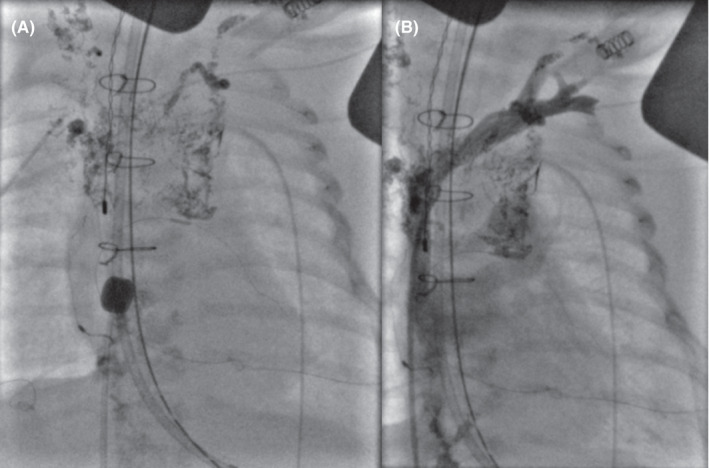
(A) Catheterization with conventional lymphangiogram demonstrating a right‐sided thoracic duct that crosses the midline at approximately the level of T3 with no egress into the innominate vein, and no connection between the thoracic duct and the brachiocephalic vein at the left venous angle. (B) Distal innominate vein injection showing a patent brachiocephalic and innominate vein but with a significant gap between the vein and thoracic duct

A 35‐week, 2.6 kg, premature male with truncus arteriosus type 2 and postnatal diagnosis of trisomy 21 underwent complete truncus repair with a RV‐PA conduit with a 9‐mm pulmonary homograft, bilateral pulmonary artery arterioplasty, VSD closure with a sauvage patch, and adjustable atrial septal defect closure on day of life 9 with delayed sternal chest closure on postoperative day (POD) 6. He was noted to have chylous chest tube output on POD 9 and was, therefore, placed on conservative dietary therapy per our institution's chylothorax protocol. Due to increasing chylous output over the subsequent 48 h, octreotide infusion was initiated, albeit with no interval improvement. In addition to significant chylous output (Figure 1), he developed associated nutritional complications namely hypogammaglobulinemia, hypoalbuminemia, and hypercoagulability. On POD 13, he underwent a diagnostic catheterization to assess the etiology of these significant chylous effusions. His cardiac hemodynamics were significant for near‐systemic RV and PA pressures. Angiography demonstrated right pulmonary artery narrowing (RPA) and no aorto‐pulmonary collaterals. The RPA was consequently stented with interval reduction in PA pressure to half systemic. Despite this significant improvement in hemodynamics, his chylous output continued to rise, reaching as high as 350 cc/kg/day. On POD 15, an intranodal dynamic contrast‐enhanced MR lymphangiogram (IN‐DCMRL) showed an abnormal macro‐lymphatic architecture. There was a diminutive, near plexiform cisterna chyli lying left of the midline with a continuous thoracic duct also lying to the left of the midline and then immediately crosses to the right aspect of the spine and ascending along the right of the spine with numerous cystic dilatations and several foci of contrast leak in the right and left pleural cavities. The thoracic duct was noted to again cross the midline at T5 to its terminus at the left subclavian/innominate vein confluence (Figure 2A). A 3D reconstruction of the lymphatic channels was additionally performed for better understanding and visualization of his complex anatomy (Figure 2B). He subsequently underwent an intranodal lymphangiogram on POD 18, which revealed no obvious cisterna chyli target for percutaneous transabdominal access, complete opacification of the TD with abnormal tracts into the bilateral lungs, and no connection to the innominate vein (Figure 3). The proximal TD was unable to be accessed, and given the aforementioned anatomy, the decision was made to proceed conservatively with a TD disruption below the cisterna chyli using the access 22‐gauge Chiba needle and IN‐Lipiodol contrast infusion. This was done to decrease the volume of chylous loss given the high‐volume output for this sized infant. Fluoroscopy showed no further movement of lipiodol to the TD and confirmed a procedural success. The procedure yielded modest clinical effect, temporarily reducing chylous output to <120 cc/kg/day. However, his post‐procedure course was complicated by septic shock with a resulting uptrend in his chylous output peaking to 260 cc/kg/day. He additionally required pericardiocentesis with the placement of a pericardial drain for chylopericardium and initiation of peritoneal dialysis for chylous ascites and renal failure.

**FIGURE 4:**
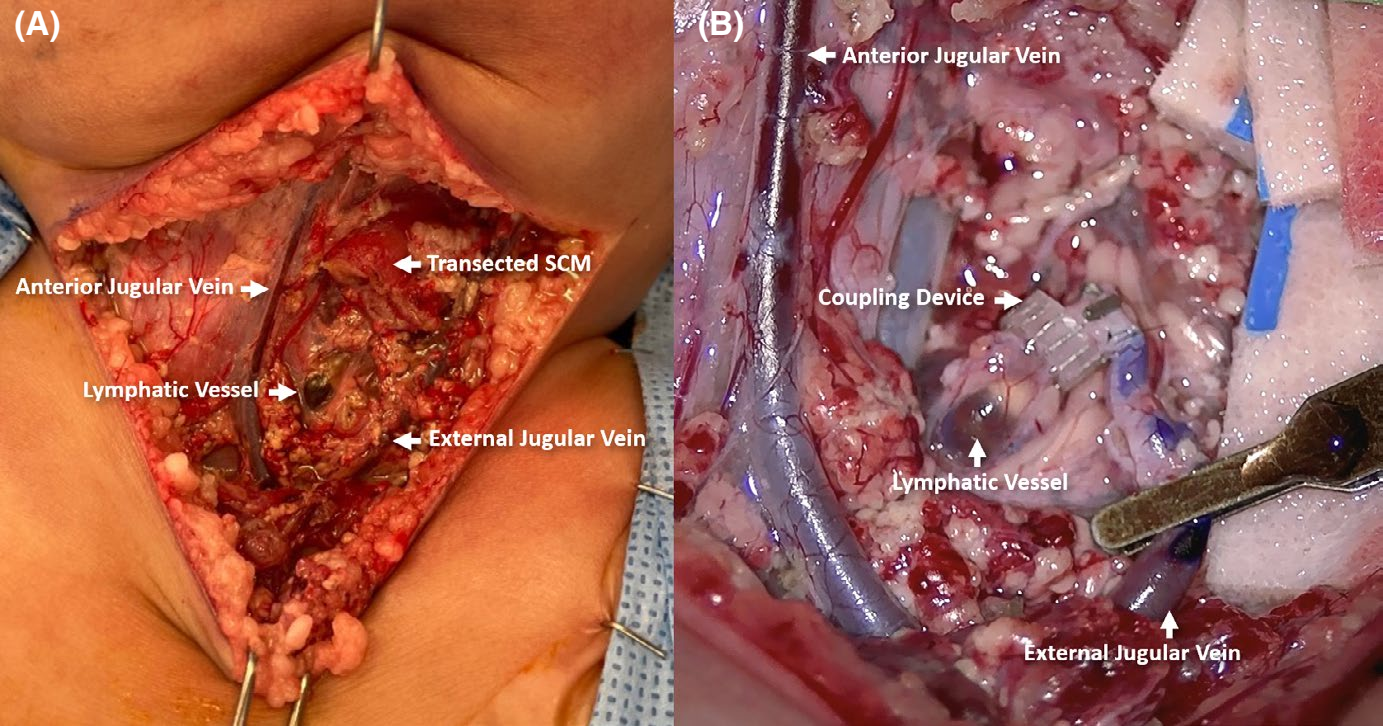
(A) Neck dissection revealing local anatomy including the transected left sternocleidomastoid (SCM) muscle with direct visualization of the anterior jugular vein, external jugular vein, and the thoracic duct (lymphocele with methylene blue dye that was injected into inguinal notes). (B) Microvascular reconstruction of the thoracic duct with left neck lymphovenous anastomosis involving the thoracic duct and the left external jugular vein using a 2‐mm Synovis coupling device

After clinical stabilization, he underwent multidisciplinary microvascular lymphovenous anastomosis with head and neck surgery, plastic surgery, and interventional cardiology on POD 42 given his complex lymphatic anatomy and the above‐stated findings. At the start of the procedure, a lymphangiogram was performed by injecting methylene blue into the inguinal lymph nodes; 1 h was allowed for the lymphatic dye to course through the lymphatic system for optimal visualization of the neck vessels. Prior to incision, ultrasound guidance was utilized in the identification and mapping of the venous structures in the neck. Neck dissection was then performed by head and neck surgery. The left anterior jugular vein and the left external jugular vein were dissected toward the subclavian vein. The sternocleidomastoid was divided to optimize exposure. During dissection, chyle was noted to be spilling throughout the surgical bed. A large dilated lymphatic channel was identified upon entering the carotid sheath. The left external jugular was clipped superiorly and rotated to form a gentle loop to the lymphatic channel. A microvascular anastomosis was completed using a 2‐mm Synovis coupling device (Synovis Life Technologies; Figure 4). Initially, there was reflux of venous blood into the lymphatic system, but after observing for 5 min in the operating room, the flow changed from lymphatic to venous direction. His immediate postoperative course was complicated by reversal of flow leading to venous to lymphatic drainage and resulting sanguineous chylous output requiring hemodynamic support and elevation of the head of bed to lower CVP, which quickly re‐established antegrade lymphatic to venous flow.

The week following the procedure, chest tube output gradually decreased to an eventual 0 cc. The decrease in chylous output from the chest tubes coincided with a paradoxical increase in peritoneal output. He continued to require peritoneal dialysis, which was later transition to continuous renal replacement therapy (CRRT) for refractory chylous ascites and ongoing renal failure; he additionally remained in critical condition requiring significant vasoactive and ventilatory support. Due to a significant episode of profound irreversible bradycardia refractory to transesophageal pacing, CPR and multiple doses of calcium, bicarbonate, and epinephrine, the patient died on POD 61 (DOL 70).

## 3 DISCUSSION

Conservative management of chylothorax and lymphedema includes low‐fat/no‐fat dietary therapies, chest tube placement, and medications like octreotide that have been reported to resolve up to 70% of cases. However, mortality rates rise significantly to 34% when conservative management fails.^2^, ^5^ The range of therapies following conservative treatment failure varies widely, and none have been reliably efficacious.^3^ Not surprisingly, treatment options are undergoing rapid revisions and changes. Previous approaches utilizing surgical techniques such as pleurodesis, creation of pleuroperitoneal shunts, and surgical TD ligation are no longer employed routinely.^1^, ^6^ Currently, the use of advanced imaging techniques, specifically the use of dynamic contrast‐enhanced MR lymphangiogram (DCMRL), allows for clear visualization of the lymphatic system and permits distinguishing traumatic from non‐traumatic lymphatic malformations.^1^ Additionally, techniques such as intranodal lymphangiography and TD embolization using ethiodized oil (Lipiodol) have further advanced this emerging field but again their utilization, timing, and outcomes remain limited in literature in the management of infants with complex lymphatic disease processes.^1^, ^5^, ^7^ Of particular interest are patients categorized with central lymphatic flow disorder (CLFD) as in the case above. In the previous case series, CLFD has a mortality rate greater than 50%.^1^, ^5^ CLFD has been shown to be difficult to treat with catheter‐based lymphatic interventions, and there are mixed outcomes in improving lymph burden without a significant trend toward survival.^1^, ^5^ Of the very limited literature and data available, it appears that LVA gives these vulnerable patients the best chance of resolution of chylothorax and survival to discharge.^1^, ^2^, ^5^

The case above illustrates a novel and advanced surgical technique for the use of intractable chyle leak despite percutaneous lymphatic intervention and TD disruption. This technique involved the use of a Synovis coupling device for an LVA in neonates, which allows for a rapid creation of the LVA, without the need of an end‐to‐end or end‐to‐side microsurgical suturing technique. Previously published reports of LVA surgeries in neonates describe hand‐sewn end‐to‐end or end‐to‐side thoracic duct to venous anastomosis.

The creation of an LVA is a complex undertaking that requires extensive multidisciplinary conversations, cooperation, and coordination. Significant clinical expertise is needed to perform appropriate lymphangiography for clear surgical identification of the lymphatic channels, extensive neck and carotid sheath dissection for delineation of venous structures, meticulous microvascular techniques, and finally extensive pre‐ and postoperative critical care management. Our ad hoc creation of a comprehensive surgical lymphatic team was brought on by the need for additional treatment following the thoracic duct disruption.

Initially, the postoperative course was complicated by postoperative vasoplegia and low cardiac output creating high CVP causing backflow of venous blood to the lymphatics and resulted in mixed chylous and sanguineous output in all drains. Augmentation of cardiac output, dialysis, and elevation of the head of the bed allowed for quick re‐establishment of antegrade flow from lymphatics to venous. However, ongoing care required a difficult balancing act to facilitate antegrade LVA flow despite arrhythmic cardiac complications in the setting of anuric renal failure. In future, using a vein with functional valves distal to the anastomosis site may be crucial to help prevent the reversal of flow complication as seen in recent reports.^8^ Moreover, once the diagnosis of an obstructed TD is made via IN‐DCMRL, the clinical decision‐making should be to proceed immediately with a surgical LVA. The effort seen here to perform a conservative percutaneous intervention with TD disruption, and lipiodol embolization was performed in an effort to buy time and temporize the chylous losses while the feasibility of performing an LVA at our center was investigated.

When to consider an LVA versus percutaneous lymphatic embolization can be difficult given the limited experience and practice standards.^2^ Therefore, Salva et al highlight the use of DCMRL imaging to characterize central lymphatic abnormalities in order to predict the type of interventions that will provide the most benefit to a particular patient.^1^, ^3^ Now after the creation of a lymphatics team capable of successfully performing LVA, the authors would advocate for proceeding directing to LVA in future cases of CFLD at our institution. Ultimately, further research and advancement in the field is needed to guide treatment for these highly complex lymphatic cases.

## 4 CONCLUSION

LVA is a novel surgical treatment for intractable neonatal chylothorax, and additional literature is needed to identify proper patient selection, timing, and suitability. It requires multidisciplinary care and planning for treatment success.

## CONFLICT OF INTEREST

The authors of this manuscript declare no conflict of interest.

## AUTHOR CONTRIBUTIONS

JR wrote the manuscript. MH obtained the figures and tables. GS and SS revised the manuscript, figures, and tables.

## ETHICAL APPROVAL

This manuscript was completed in accordance with the ethical standards of the institutional research committee.

## CONSENT

Written informed consent was obtained from the patient's parents to publish this report in accordance with the journal's patient consent policy.

## Data Availability

None.
